# PagARF3.1 promotes adventitious root formation by repressing *IPT*-mediated cytokinin biosynthesis

**DOI:** 10.48130/forres-0025-0018

**Published:** 2025-08-29

**Authors:** Ying-Li Liu, Xue-Qin Song, Hui He, Meng-Xuan Hu, Meng-Zhu Lu, Shu-Tang Zhao

**Affiliations:** 1 State Key Laboratory of Tree Genetics and Breeding, Chinese Academy of Forestry, Beijing 100091, China; 2 State Key Laboratory of Tree Genetics and Breeding, Research Institute of Forestry, Chinese Academy of Forestry, Beijing 100091, China; 3 Co-Innovation Center for Sustainable Forestry in Southern China, Nanjing Forestry University, Nanjing 210037, China

**Keywords:** Adventitious roots, Auxin signaling, Cytokinin, *PagARF3.1*, *PagIPTs*, *Populus*

## Abstract

Adventitious root formation is a crucial developmental process in the clonal propagation of economically significant horticultural and woody species. This process is antagonistically regulated by auxin and cytokinin, yet the underlying molecular regulatory mechanisms remain poorly understood. In this study, we investigated the role of *PagARF3.1*, a homolog of *Arabidopsis* auxin response factor 3, in hybrid poplar (*Populus alba* × *Populus glandulosa* clone cv. '84K'), focusing on its involvement in adventitious root formation. GUS staining analysis revealed that *PagARF3.1* was expressed in adventitious root tips, pericycle cells, early root primordia, and outgrowing roots. Knockdown of *PagARF3.1* delayed adventitious root formation and reduced root biomass in transgenic plants, whereas overexpression of *PagARF3.1* promoted earlier rooting and increased the number of adventitious roots. Real-time quantitative polymerase chain reaction analysis indicated that the expression levels of *PagIPT5a* and *PagIPT5b* were significantly elevated in *PagARF3.1* RNAi lines and reduced in overexpression lines. Yeast one-hybrid assays and ChIP-PCR analysis demonstrated that PagARF3.1 directly binds to the promoter regions of *PagIPT5a* and *PagIPT5b*, thereby regulating their expression. These findings collectively demonstrate that PagARF3.1 acts as a positive regulator of adventitious root formation by repressing IPT-mediated cytokinin biosynthesis.

## Introduction

Root systems are fundamental to plant growth and development, performing two key functions: anchoring plants in the soil and facilitating water and nutrient uptake^[1]^. The root system of angiosperms includes the primary root, originating from the radicle, as well as postembryonically formed lateral and adventitious roots (AR)^[2,3]^. Unlike lateral roots that originate from pericycle cells adjacent to xylem poles, ARs are developed from cambial or other meristematic cells in aboveground plant tissues^[4]^. The process of adventitious rooting is a critical developmental event, particularly significant in the clonal propagation of plants. This is especially true for long-lived species with extended generation cycles, such as *Populus* and other perennial trees^[5,6]^. Consequently, the formation of ARs is a pivotal factor in the successful clonal propagation of economically valuable woody species. Despite its importance, the molecular basis of AR initiation and development from cuttings has not been fully elucidated in tree species.

AR formation has been systematically categorized into three distinct developmental phases: activation, induction, and outgrowth, based on physiological and anatomical processes^[7]^. This complex developmental program is primarily regulated by endogenous genetic mechanisms while being significantly modulated by environmental factors^[4]^. Among various phytohormones involved in AR development, auxin emerges as the principal regulatory component, exerting crucial control over multiple aspects of root formation^[8]^. For decades, exogenous auxin application has been employed to stimulate and enhance root formation in stem cuttings of recalcitrant species. In *Arabidopsis*, mutants overproducing free and conjugated indole-3-acetic acid (IAA) developed excess ARs on the hypocotyls^[9]^. On the other hand, treatment of auxins at AR formation phases severely inhibited adventitious rooting in poplar stem cuttings^[10]^. In numerous plant species, the localized accumulation of auxins in cutting bases enables specific cells to acquire root-forming competence, initiating the genetic program for root organogenesis^[11]^. These collective findings underscore auxin's central position in the hormonal network governing AR development, while simultaneously highlighting the complexity of its mode of action across different species and developmental contexts.

Auxin plays multifaceted roles in AR formation, orchestrating processes ranging from cellular fate determination to the initiation, emergence, and subsequent development of root primordia^[1]^. Emerging evidence across plant species indicates that IAA promotes AR formation through activation of auxin signaling pathways and modulation of auxin-responsive gene expression, mirroring mechanisms observed in lateral root development^[12,13]^. The core auxin signaling network in plants consists of three key protein families: TIR1/AFB receptors, Aux/IAA repressor proteins, and auxin response factors (ARFs)^[14]^. Functioning as auxin receptors, TIR1/AFB F-box proteins mediate the degradation of Aux/IAA proteins, thereby releasing ARFs from transcriptional repression^[15,16]^. These liberated ARFs subsequently regulate downstream gene expression, driving ARF-dependent auxin responses. The ARF family exhibits significant genetic diversity across plant species, with *Arabidopsis* and *Populus* containing 23 and 39 identified members, respectively^[17,18]^. Research in *Arabidopsis* hypocotyls demonstrates that ARF transcript balance critically regulates AR initiation. ARF6 and ARF8 positively regulate developmental processes, in contrast to ARF17, which exerts negative regulation^[12]^. Intriguingly, this regulatory paradigm shows species-specific variation. In hybrid poplar (*Populus davidiana × Populus bolleana*), *PeARF17* promotes AR development, contrasting with its inhibitory role in Arabidopsis^[19]^.

Cytokinin exhibits antagonistic effects against auxin throughout various stages of AR formation. During AR formation, auxin concentrations are highest in the initiation phases but decline as roots emerge, in contrast to cytokinins, which demonstrate an inverse pattern of accumulation^[20]^. Acting as a negative regulator, cytokinin suppresses AR formation across multiple plant species. Exogenous cytokinin application during the induction phase significantly inhibits root initiation in cuttings^[20]^. In addition, transgenic lines of tobacco and *Arabidopsis* where the cytokinin oxidase gene was overexpressed, the reduction in cytokinin content correlated with increased AR production^[21]^. Similarly, *Arabidopsis* mutants with impaired cytokinin biosynthesis or signaling exhibit increased AR production^[21]^. Although the hormonal crosstalk between auxin and cytokinin in AR regulation is well-established, the molecular basis of their antagonistic interaction remains unclear. In *Arabidopsis*, ARF3 modulates shoot regeneration and floral meristem determinacy by directly binding to the promoter of *AtIPT5*, a rate-limiting enzyme in cytokinin biosynthesis and suppressing its expression^[22,23]^. Notably, *AtIPT5* shows specific expression patterns in root primordia, columella, and root caps^[24]^, suggesting this auxin-cytokinin regulatory axis may influence both adventitious and lateral root development.

Our current study identifies *PagARF3.1* from hybrid poplar (*Populus alba × Populus glandulosa* clone cv. '84K') as a positive regulator of AR formation. We provide mechanistic evidence that PagARF3.1 directly binds to *IPT* gene promoters to modulate their expression. This regulatory interaction plays an essential role in sustaining auxin-cytokinin balance during *de novo* AR regeneration.

## Materials and methods

### Plant materials and growth conditions

The hybrid poplar clone 84K was maintained in a tissue culture room under controlled conditions: 23−25 °C with a 16 h light/8 h dark cycle on half-strength Murashige and Skoog (1/2 MS) nutrient medium. *PagARF3.1* promoter::GUS transgenic plants were used to monitor the expression of *PagARF3.1* during AR formation. For AR induction experiments, leafy stem segments were collected from three sources: 3-week-old seedlings of *PagARF3.1* overexpression (OE) lines, RNA interference (RNAi) transgenic lines, and wild-type (WT) 84K controls. Clonally propagated OE and RNAi plants were subsequently acclimatized in soil and grown for three months under greenhouse conditions at the Chinese Academy of Forestry (Beijing, China).

### RNA isolation and real-time quantitative polymerase chain reaction analysis (RT-qPCR)

The RNeasy Plant Mini kit (Qiagen) was utilized for total RNA extraction. For cDNA synthesis, 1 μg of purified RNA was reverse transcribed using the First Strand cDNA Synthesis kit (Thermo Scientific, USA) according to the manufacturer's instructions. The specific primers used for RT-qPCR are listed in Supplementary Table S1. *PagACTIN* served as the reference gene to normalize cDNA levels across different tissues and genotypes in poplar. All experiments were performed with at least three independent biological replicates.

### Plasmid construction and genetic transformation

A 2.1 kb fragment containing the 5′-UTR region of *PagARF3.1* was amplified from 84K poplar genomic DNA and inserted into a pMDC164 vector to produce the *proPagARF3.1::GUS* reporter construct. The complete coding region of *PagARF3.1* was isolated from cDNA and ligated into pMDC32, producing a constitutive 35S::*PagARF3.1* overexpression vector. For RNA interference, a 411 bp coding region fragment of *PagARF3.1* was cloned in both sense and antisense orientations into the pBI121 vector, producing the *35S::PagARF3.1-RNAi* construct. All plasmid constructs were validated by sequencing and were transformed into *Agrobacterium* GV3101 by electroporation. All the primers are listed in Supplementary Table S1. Transgenic poplars were created through *Agrobacterium tumefaciens* transformation according to previously established procedures^[25]^. Transformants were identified on selective medium with 3 mg/L hygromycin for *proPagARF3.1::GUS* and *35S::PagARF3.1* or 50 ng/L kanamycin for *35S*::*PagARF3.1-*RNAi. For each construct, more than 10 independent transgenic lines were established and characterized.

### Subcellular localization

Protein localization studies were carried out according to previously published methods^[25]^. Four-week-old leaves were injected with *Agrobacterium tumefaciens* strain GV3101 containing the *35S::PagARF3.1-GFP* vector. After inoculation, plants were kept in darkness for 12 h and subsequently cultivated under controlled environmental conditions for 3 d. Before imaging, leaves were stained with 50 μM 4',6-diamidino-2-phenylindole (DAPI) for 10 min. Fluorescence imaging was performed on an Ultra VIEW VoX3D Live Cell Imaging System (PerkinElmer, USA), utilizing 488 nm excitation for GFP visualization and 405 nm for DAPI detection.

### GUS staining

Histochemical GUS staining was conducted following the protocol of Yuan et al.^[25]^. The samples underwent fixation in 90% acetone at 0−4 °C for 30 min, followed by three washes with rinse solution [50 mM sodium phosphate (pH 7.0), 0.1% (v/v) Triton X-100, 2.5 mM potassium ferrocyanide, and 2.5 mM potassium ferricyanide]. For GUS staining, tissues were treated with a buffer containing 1 mM X-Gluc, vacuum-infiltrated (20 min, RT), and then incubated at 37 °C in the dark for 6−8 h. Finally, ethanol (70%) was used to clear the samples before visualization. For detailed spatial analysis of GUS activity, cross-sections were prepared from the basal 3 mm of stem cuttings (AR initiation zone) harvested at 0, 3, 4, and 6 d after the induction of AR, using the protocol of Zhang et al.^[26]^. Sections (50 μm thickness) were generated using a VT1000S vibratome (Leica, Wetzlar, Germany), fixed in 90% acetone under ice-cold conditions for 30 min before GUS staining. The cleared tissue sections were examined using an Axio Imager A1 microscope (Zeiss, Germany). Three independent biological replicates from promoter::GUS transgenic lines were analyzed per time point. Each experiment was repeated three times with consistent results.

### Exogenous auxin and cytokinin treatment

Referencing *Arabidopsis*-related methods^[27]^, WT and *PagARF3.1* overexpressing transgenic poplar were selected. The 2^nd^ and 3^rd^ leaves from the top of each plant were collected, and half of each leaf was used after removing the petiole. The prepared leaf segments were cultured on MS medium supplemented with varying concentrations of phytohormones. For auxin treatment, three types of culture media were set up: 0 mg/L IAA, 1 mg/L IAA, 1 mg/L IAA + 10 µM NPA. The leaves were cultured on the corresponding media for two weeks. For cytokinin treatment, MS culture media supplemented with 0 µg/L 6-BA, 0.5 µg/L 6-BA, and 1.5 µg/L 6-BA were used, respectively, with the same two-week cultivation period. Following culture, ARs inducted from leaf explants were carefully examined and quantified.

### Cytokinin content determination

Stem segments from one-month-old WT and *PagARF3.1*-overexpressing plants were selected. The cytokinin content in these samples was measured using liquid chromatography-tandem mass spectrometry (LC-MS/MS).

### Yeast one hybrid

Protein-DNA binding interactions were examined using yeast one-hybrid (Y1H) assays (K1617-1, Takara Bio, Palo Alto, CA, USA). The promoter sequences of *PagIPT5a* and *PagIPT5b* were isolated using PCR amplification and inserted into the pHIS2 cloning vector. To construct AD-PagARF3.1, the coding region of *PagARF3.1* was ligated into the pGADT7-Rec2 vector. The assays were performed following the manufacturer's procedures. Primer sequences are provided in Supplementary Table S1.

### Chromatin Immunoprecipitation-PCR (ChIP-PCR)

ChIP-PCR assays were conducted following established protocols^[28]^. The coding sequence of *PagARF3.1* was ligated in-frame with an N-terminal FLAG epitope tag, which was subsequently inserted into the pBI121 plant expression vector downstream of the constitutive CaMV 35S promoter. For ChIP-PCR analysis, sonicated chromatin samples were prepared and divided into three groups: input (sonicated chromatin before immunoprecipitation), mock (chromatin immunoprecipitated without the anti-FLAG antibody), and anti (chromatin immunoprecipitated with the anti-FLAG antibody). *PagACTIN1* was used as an internal control to normalize the data. The experiments were conducted with three independent biological replicates to ensure repeatability and consistency of the results.

## Results

### Characterization and comparative sequence analysis of *AtARF3* orthologous genes in *Populus*

*Populus* has three putative orthologs of *Arabidopsis ARF3*, *PtrARF3.1*, *PtrARF3.2,* and *PtrARF3.3*. *PtrARF3.3* is a pseudogene^[18]^. Therefore, two putative *ARF3* genes were cloned from the hybrid aspen clone 84K and named *PagARF3.1* and *PagARF3.2*. Unlike typical ARF protein structure, which contains N-terminal B3-like DNA-binding domain (DBD), C-terminal dimerization (CTD) domain and a variable middle region (ARF), both PagARF3.1 and PagARF3.2 contain only two conserved domains: DBD domain and ARF domain, indicating that PagARF3s miss the function of interaction with other Aux/IAA or ARF proteins (Supplementary Fig. S1). PagARF3.1 and PagARF3.2 share 85.86% amino acid sequence identity and 59.29% and 61.04% identity with AtARF3, predicted to function as a transcriptional repressor.

### *PagARF3.1* expression pattern and subcellular localization

**Figure 1:**
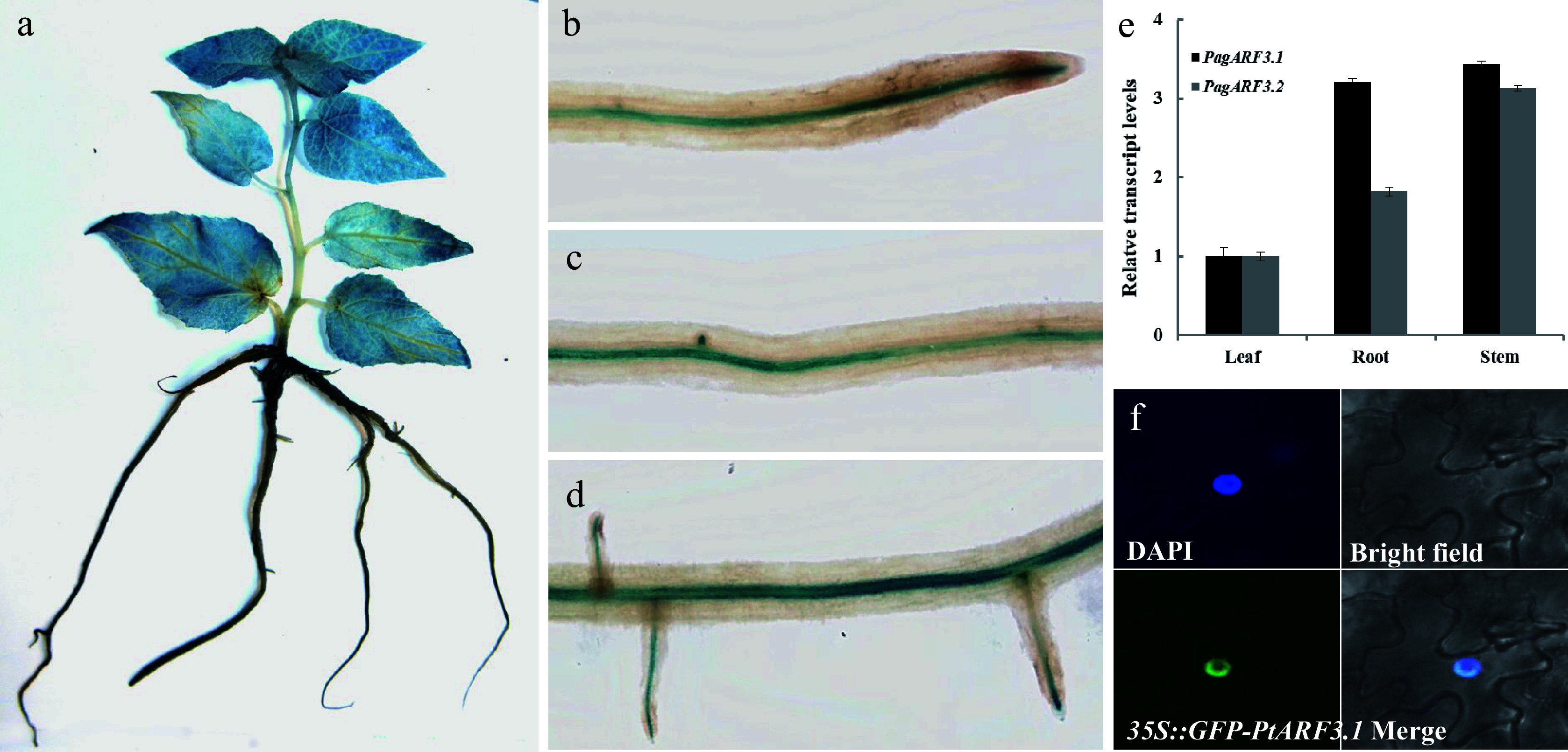
Expression patterns and subcellular localization of *PagARF3.1*. GUS-staining assays of *PagARF3.1* in (a) 3-week-old sapling and (b)−(d) root. (e) The expression of *PagARF3.1* in roots, leaf, and stem. (f) The nuclear localization of PagARF3.1.

To examine the tissue-specific expression profiles of *PagARF3.1* and *PagARF3.2* in poplar, we analyzed their transcript levels across various tissues using RT-qPCR. The results revealed distinct expression profiles: *PagARF3.2* exhibited preferential expression in stems, whereas *PagARF3.1* showed high expression levels in both stems and roots (Fig.1e). Based on this broader expression pattern, *PagARF3.1* was selected for further characterization. To delineate the spatial expression of *PagARF3.1* in greater detail, we generated transgenic 84K poplar lines expressing a *proPagARF3.1::GUS* reporter construct. Histochemical GUS staining demonstrated widespread *PagARF3.1* expression in leaves, young stems, and roots (Fig. 1a), which was consistent with the RT-qPCR data. Higher-resolution analysis of root tissues revealed GUS activity in the apical regions of ARs, pericycle cells, early lateral root primordia, and emerging lateral roots (Fig. 1b–d). These observations suggested stage-specific regulation of *PagARF3.1* during root development, implicating its potential functional role in this process.

To determine the intracellular distribution of PagARF3.1, we performed transient expression assays in *Nicotiana benthamiana* leaf epidermal cells using a C-terminal GFP fusion protein (PagARF3.1-GFP). Fluorescence microscopy revealed exclusive nuclear localization of the fusion protein, demonstrating complete co-localization with the nuclear marker DAPI in dual staining assays (Fig. 1f). This nuclear confinement supports the predicted role of PagARF3.1 as a transcriptional regulator.

### Dynamic expression pattern of *PagARF3.1* during AR formation

**Figure 2:**
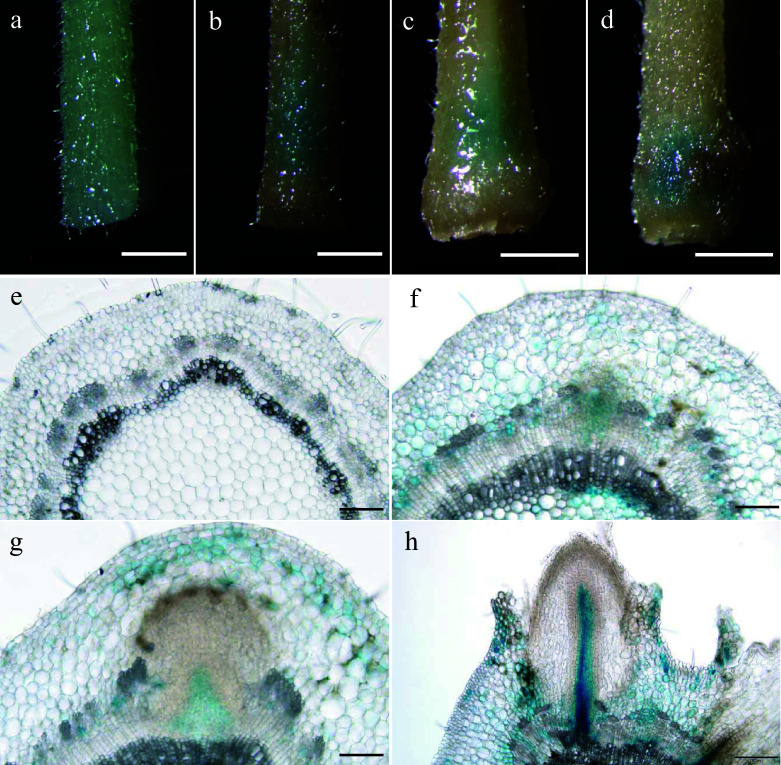
Expression patterns of *PagARF3.1* during AR formation. GUS staining of *proPagARF3.1::GUS* (a)−(d) leafy stems, and their (e)−(h) transverse sections; the samples were collected at (a), (e) 0 d, (b), (f) 3 d, (c), (g) 4 d, (d), (h) 6 d. Scale bars: (a)−(d) 1 mm; (e)−(g) 50 µm; (h), 200 µm

To decipher the biological role of *PagARF3.1* during AR formation, we performed temporal expression profiling using spatial localization analysis through histochemical staining in poplar. Our results revealed a dynamic expression pattern of *PagARF3.1* during AR formation. We conducted histochemical analysis using *proPagARF3.1::GUS* transgenic poplars throughout AR development. Before induction, GUS staining showed uniform distribution across stem sections (Fig. 2a). During days 3−4 post-induction, the staining became progressively concentrated in specific regions destined for AR emergence (Fig. 2b, c). By day 6, strong GUS activity was specifically localized to stem tissues surrounding developing ARs (Fig. 2d). Cross-sectional analysis further revealed nuanced spatial expression patterns: Pre-induction stems exhibited weak, ubiquitous GUS signals (Fig. 2e). Following induction (days 3−4), signal intensity increased markedly in AR primordia, particularly within cambial and phloem zones (Fig. 2f, g). During AR elongation at day 6, expression became restricted to pericycle cells adjacent to developing roots (Fig. 2h). This spatiotemporal expression pattern suggests that PagARF3.1 plays a critical regulatory role in early AR initiation events in poplar.

### PagARF3.1 promotes AR formation in poplar

**Figure 3:**
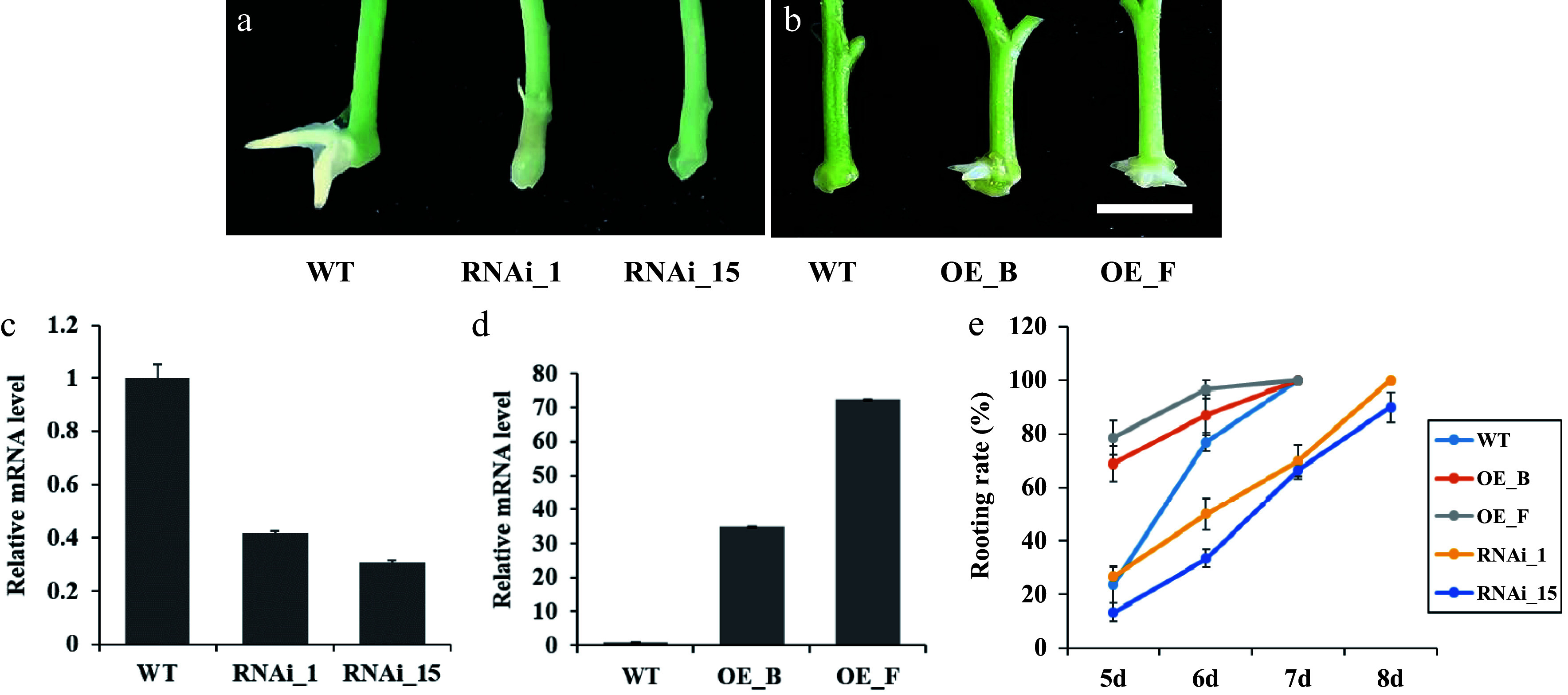
Manipulation of *PagARF3.1* expression affects the AR development of transgenic poplar. (a) Representative images of the AR formation of WT and RNAi_1 and RNAi_15 after 7 d excision. (b) Representative images of the AR formation of WT and OE_B and OE_F after 5 d excision. Bars = 5 mm. (c) RT-qPCR analysis of *PagARF3.1* transcripts in the WT and overexpression lines. (d) RT-qPCR analysis of *PagARF3.1* transcripts in the wild type and RNAi lines. (e) Rooting rates of wild type, OE, and RNAi lines.

To further understand the function of *PagARF3.1* in AR regeneration, we engineered transgenic poplar lines with RNA interference-mediated knockdown (RNAi) and constitutive overexpression (OE) of this gene. RT-qPCR analysis identified 10 independent RNAi lines showing significantly reduced expression and 12 OE lines with elevated *PagARF3.1* mRNA levels. Two representative lines of each RNAi (RNAi_1 and RNAi_15) or OE (OE_B and OE_F) transgenic plants were selected for further analysis (Fig. 3c, d). The emergence of ARs was delayed in *PagARF3.1* RNAi lines compared to the WT (Fig. 3a). In contrast, the OE lines exhibited earlier root emergence than the WT (Fig. 3b). In general, AR emergence of WT plants was typically observed on the sixth day after stem segments were excised and placed in tissue culture medium. However, in the RNAi lines, only about 30% of the stem segments developed ARs by the sixth day, with this percentage increasing to 80% by the eighth day after excision. Conversely, the rooting rate of *PagARF3.1* overexpressors reached 70%−75% by the fifth day, and 100% by the sixth day after stem excision (Fig. 3e).

**Figure 4:**
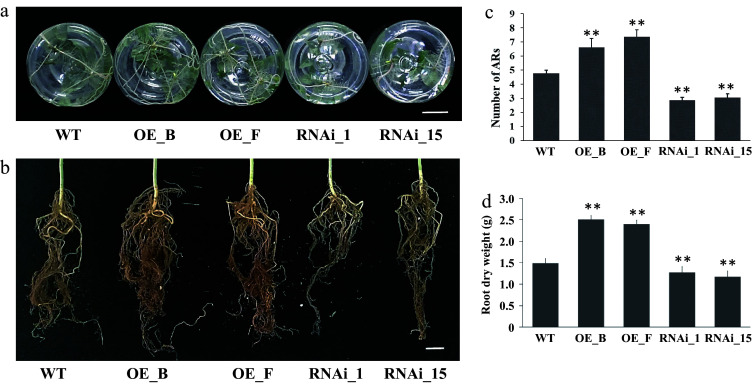
ARs from leafy stems of *PagARF3.1* RNAi lines, OE lines, and WT. (a) AR systems of WT, OE, and RNAi line after 4 weeks of transfer to rooting induction medium. (b) AR system from 3 month old plants in soil. (c) Number of adventitious roots per cutting in WT, OE, and RNAi line. (d) The dry weight of ARs from 3 month old plants. Bars = 2 cm. Significant differences between WT and transgenic lines are indicated with asterisks. Significance analysis was performed by Student's t-test (** *p* < 0.01).

Quantitative analysis of AR production further revealed that 4-week-old seedlings of the three genotypes exhibited significant differences in AR numbers. On average, WT 84K seedlings formed 4.77 ± 0.23 (SE) roots per cutting. In contrast, the RNAi lines (RNAi_1 and RNAi_15) formed 2.87 ± 0.19 and 3.07 ± 0.26 roots per cutting, respectively. The OE lines (OE_B and OE_F) exhibited a higher number of ARs, generating 6.63 ± 0.19 and 7.37 ± 0.26 roots per cutting, respectively (Fig. 4a, c). Additionally, the root dry weight of RNAi and OE lines was significantly decreased or increased compared to WT seedlings after 3 months of cultivation in soil (Fig. 4b, d). These results collectively suggest that *PagARF3.1* positively regulates AR regeneration.

### PagARF3.1 regulates AR formation by enhancing auxin signaling and suppressing cytokinin signaling

To test whether *PagARF3.1* promotes AR formation via the auxin pathway, leaves from *PagARF3.1* ectopic OE lines were treated with auxin. After two weeks of culture in auxin-free medium, WT leaves rarely formed ARs. In contrast, nearly 50% of *PagARF3.1* OE leaves produced ARs, with a significant increase in root number (Supplementary Fig. S2a, d, e). When the medium was supplemented with 1 mg/L IAA, the rooting rate of control lines reached 63%, whereas approximately 83% of *PagARF3.1* OE leaves formed 2−3 ARs per leaf (Supplementary Fig. S2b, d, e). Further tests with 1 mg/L IAA and 10 µM NPA reduced the WT rooting rate to 6.7%, while *PagARF3.1* OE lines still maintained a 33% rooting rate (Supplementary Fig. S2c, d, e). These results suggest that *PagARF3.1* promotes AR formation by enhancing auxin signaling.

**Figure 5:**
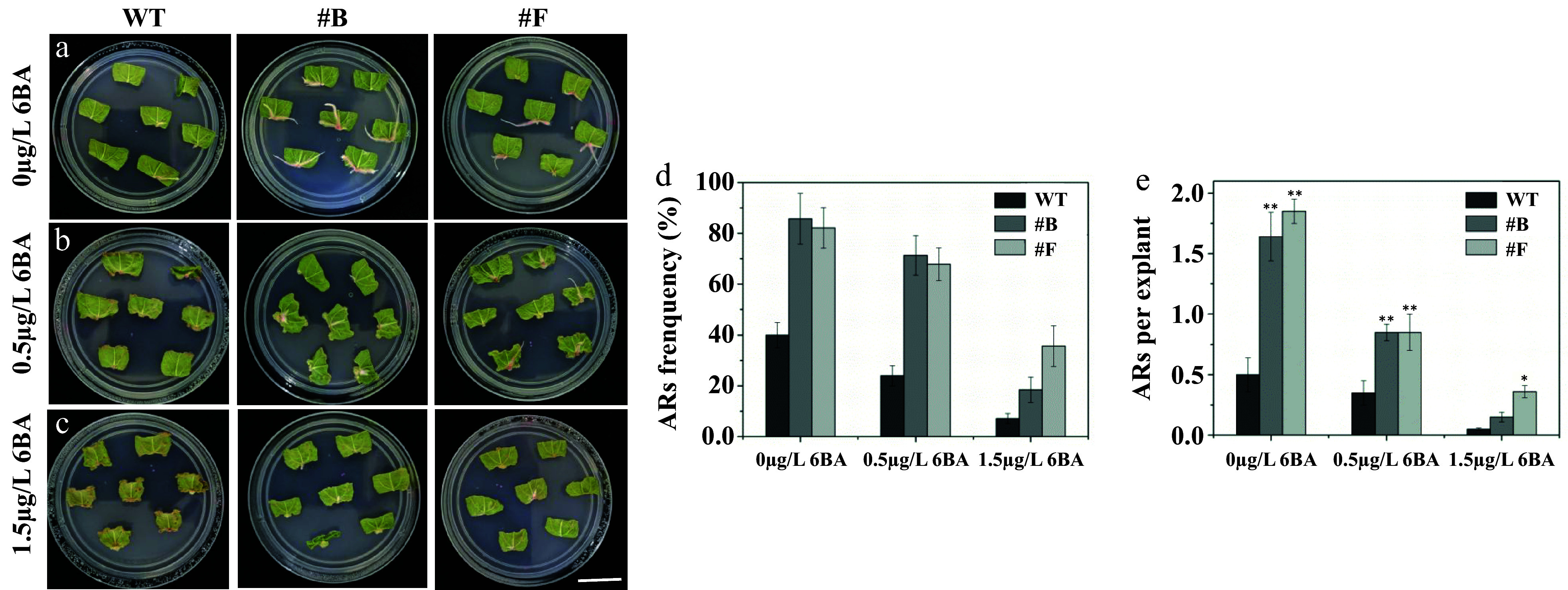
*PagARF3.1* promotes AR formation via the cytokinin pathway. Comparison of AR formation from leaf explants between the WT and the overexpression line (#B, #F) of *PagARF3.1* treated with (a) 0 µg/L 6-BA, (b) 0.5 µg/L 6-BA. (c) 1.5 µg/L 6-BA. Comparison of (d) AR formation frequency, and (e) the numbers of AR. Significance analysis was performed by Student's t-test (* *p* < 0.05 and ** *p* < 0.01). Bar = 2 cm.

Using the experimental approach, cytokinin was applied to *PagARF3.1* transgenic poplar leaves to assess its effect on AR formation. After two weeks of culture in cytokinin-free medium, the rooting rate of *PagARF3.1* OE lines reached 85%, significantly higher than that of WT plants (40%) (Fig. 5a, d, e). When the medium was supplemented with 0.5 µg/L 6-benzylaminopurine (6-BA), the WT rooting rate was 24% (Fig. 5b, d, e), whereas approximately 71% of *PagARF3.1* OE leaves formed 1−2 ARs per leaf. At a higher concentration of 1.5 µg/L 6-BA, WT lines showed negligible root formation, yet *PagARF3.1* OE lines maintained an 18% rooting rate (Fig. 5c−e). These results indicate that *PagARF3.1* promotes AR formation through inhibition of cytokinin signaling.

### PagARF3.1 negatively regulates *PagIPT5a* and *PagIPT5b* expression during adventitious root formation

**Figure 6:**
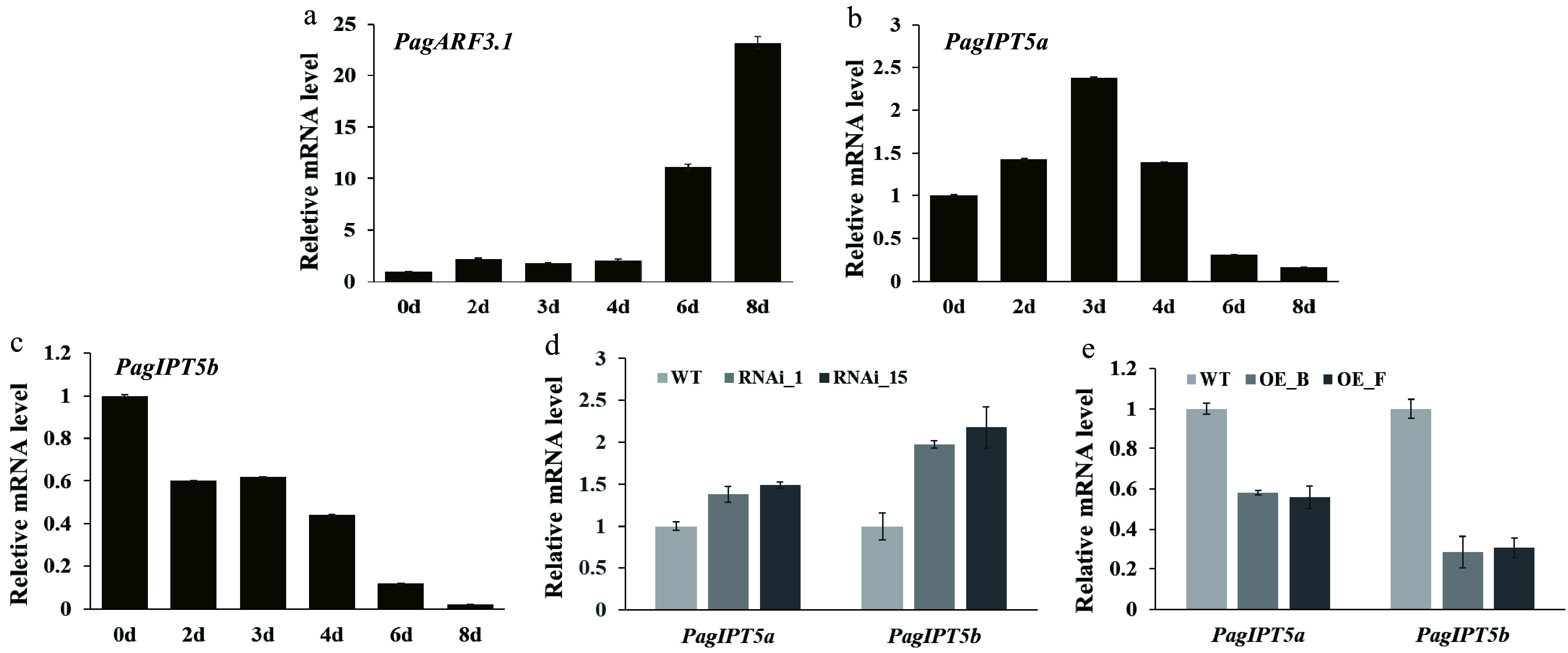
PagARF3.1 negatively regulates *PagIPT5a* and *PagIPT5b* expression. (a)−(c) RT-qPCR analysis of *PagARF3.1*, *PagIPT5a,* and *PagIPT5b* during AR induction. (d), (e) *PagIPT5a* and *PagIPT5b* expression levels in PagARF3.1 RNAi and OE lines.

In *Arabidopsis*, ARF3 is known to directly repress *IPT* gene expression to regulate *de novo* shoot regeneration and floral meristem determinacy. This functional relationship led us to explore whether PagARF3 controls AR formation through the regulation of *IPT* genes in *Populus*. The *Populus* genome contains nine members of the *IPT* family, similar to *Arabidopsis*, including seven ATP/ADP isopentenyltransferase genes (one ortholog for *IPT3*, two orthologs each for *IPT5*, *IPT6*, and *IPT7*) and two tRNA isopentenyltransferases (one ortholog each for *IPT2* and *IPT9*)^[29]^. Since isopentenylated tRNAs are generally not considered the main source of cytokinins in plants^[24]^, the expression patterns of the seven ATP/ADP IPT genes were examined using RT-qPCR. Our results showed that all these genes exhibited the highest expression levels in leaves, except *PagIPT7b*, whose mRNA could not be detected in all the tissues (Supplementary Fig. S3). Notably, *PagIPT5a*, *5b*, *6a*, *6b* exhibited higher expression in roots. To investigate potential regulatory control by PagARF3.1 over these genes, we analyzed their mRNA abundance in the roots of *PagARF3.1* transgenic plants by RT-qPCR. Quantitative analysis revealed that the transcript abundance of *PagIPT5a* and *PagIPT5b* were significantly decreased in *PagARF3.1* OE lines but increased in RNAi lines (Fig. 6d, e). *PagARF3.1* expression was significantly upregulated starting at day 2 post-induction, with transcript levels increasing dramatically to over 10-fold and 20-fold of baseline values by day 6 and day 8, respectively, compared to pre-induction levels (day 0) (Fig. 6a). To further investigate the dynamic expression patterns of *PagIPT5a* and *PagIPT5b* during AR development, RT-qPCR analysis was performed during progressive stages of AR formation. The results showed that *PagIPT5a* and *PagIPT5b* had an opposite expression pattern with *PagARF3.1* (Fig. 6b, c). The cytokinin measurement results showed that compared to the WT, the content of cytokinins (TZR/DZR/trans-Zeatin/IP/IPR) was significantly reduced in *PagARF3.1* OE plants (Supplementary Table S2). All these results indicate that *PagIPT5a* and *PagIPT5b* are negatively regulated by *PagARF3.1*.

### PagARF3.1 represses *PagIPT5a* and *PagIPT5b* expression through directly binding the promoter regions

**Figure 7:**
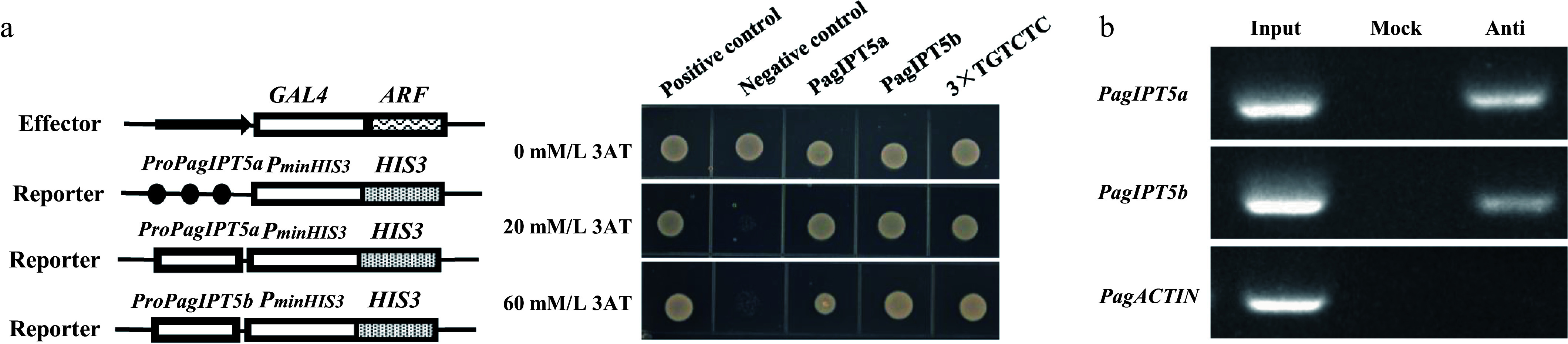
PagARF3.1 directly binds to the promoters of *PagIPT5a* and *PagIPT5b*. (a) Yeast one-hybrid analysis, and (b) ChIP-PCR analyses showed that PagARF3.1 can directly bind to the *PagIPT5a* and *PagIPT5b* promoters. Input, chromatin preparation before immunoprecipitation. Mock, immunoprecipitated without anti-FLAG antibody. Anti, immunoprecipitated with anti-FLAG antibody.

To investigate whether PagARF3.1 directly interacts with the promoter regions of *PagIPT5a* and *PagIPT5b* to modulate their transcriptional activity, we conducted yeast one-hybrid assays. In plants, ARF transcription factors are known to specifically recognize auxin response elements (AuxREs) containing the TGTCTC motif in target gene promoters. Promoter sequences spanning approximately 2,100 bp upstream of *PagIPT5a* and *PagIPT5b* were analyzed, and two AuxREs were found in each promoter. Three reporter constructs (*pHIS2-3*×TGTCTC, *pHIS2-PagIPT5a*, *pHIS2-PagIPT5b*) were generated through constructing auxin response element TGTCTC (3×) and about 200 bp fragments containing TGTCTC cis elements from each promoter of *PagIPT5a* and *PagIPT5b* into pHIS2, respectively. Each of these three reporters was co-transferred with the pGADT7-Rec2-PagARF3.1 vector into the yeast strain Y187. Positive clones were screened on SD/-leu/-trp and SD/-his/-leu/-trp medium with varying concentrations of 3-AT. Three days after transformation, only the positive control and the yeast containing pHIS2-3×TGTCTC and *pHIS2-PagIPT5a*, *pHIS2-PagIPT5b* vectors could grow normally (Fig. 7a). To confirm the direct binding of PagARF3.1 to *PagIPT5a* and *PagIPT5b*, a ChIP-PCR assay was performed. Compared to the negative control, the promoter fragments of *PagIPT5a* and *PagIPT5b* were detected in the ChIP sample, but not in the mock (Fig. 7b). Together, these results confirm that PagARF3.1 directly binds to the *PagIPT5a* and *PagIPT5b* promoters to modulate their expression.

## Discussion

AR formation is a developmental program influenced by a variety of exogenous and endogenous factors, which is crucial for clonal propagation of plants, especially for long-lived woody plants, like poplar. Thus, elucidating the regulatory mechanisms of AR formation is essential for both basic plant biology and the application of clonal propagation in commercial woody plants. In this study, we demonstrated that PagARF3.1, an auxin response factor, positively regulates AR formation in poplar stem cuttings by directly repressing *IPT* gene expression. This finding reveals a novel mechanism through which auxin-cytokinin crosstalk governs AR development.

Among all the exogenous and endogenous regulators of AR formation, auxin is a central player and exerts pleiotropic effects through multiple mechanisms including its biosynthesis, metabolic conversion, polar transport, and signal transduction pathways^[30]^. Previous studies have identified that the auxin signaling pathway is required during the AR development process. In poplar, PagFBL1, the ortholog of auxin receptor TIR1, promotes adventitious rooting through interaction with PagIAA28, though the interacting ARF remains unidentified^[31]^. ARFs, which are key regulators of the auxin signaling pathway, act as crucial functions involved in AR formation^[12,13]^. Genetic analyses have revealed that individual ARF family members mediate specific developmental programs through discrete regulatory mechanisms^[13]^. In *Arabidopsis*, *ARF6* and *ARF8* exhibit a specific function in promoting adventitious rooting while showing no measurable impact on lateral root length or number^[12]^. On the other hand, *ARF17* negatively regulates adventitious rooting through integrating auxin and light signaling pathways^[32]^. These results show that different members of the ARF gene family may have distinct functions through responding to different clues during AR formation. In this work, we showed that *PagARF3.1* was differentially expressed in various root developmental stages of lateral root and AR formation. In addition, reducing *PagARF3.1* expression could delay AR emergence and decrease AR number; however overexpressing *PagARF3.1* showed earlier root emergence and increased ARs, in comparison with the controls. These findings demonstrate that *PagARF3.1* plays crucial regulatory roles in the initiation of AR regeneration.

Previous studies have shown that *ARF3* plays various roles in the growth and development of *Arabidopsis*, ranging from gynoecium morphogenesis and self-recognition systems to organ regeneration, polarity determination, and floral meristem maintenance^[22,23,33]^. However, in the case of tomato, *SlARF3* RNAi plants exhibit phenotypic divergence from *Arabidopsis* mutants, particularly in floral organogenesis and development. Instead, SlARF3 appears to primarily regulate epidermal cell differentiation and trichome formation^[33]^. Combined with our results, we speculate that *ARF* orthologs in different species have evolved distinct functions in different developmental processes. Therefore, insight into ARF regulation mechanisms across plants is important for elucidating the functional diversity of ARF across land plants.

Environmentally or developmentally induced high cytokinin levels and the ratio of cytokinin to auxin in the stem base are correlated with suppressed AR formation across multiple plant species^[34,35]^. Trans-zeatin riboside (ZR), one of cytokinin species, can significantly inhibit AR formation of cucumber hypocotyls at concentrations of 2 × 10^−8^ M^[36]^. Similarly, *Arabidopsis* mutants of cytokinin deficiency or depression of the cytokinin response significantly enhance AR formation^[21]^. In addition, the transgenic *Arabidopsis* plants leading to lower levels of cytokinin show increased ARs, but not lateral roots^[21]^. All these results indicate that the cytokinin signaling pathway negatively regulates AR formation. In *Arabidopsis*, there are nine *IPT* genes involved in cytokinin biosynthesis and each is expressed in specific cell types^[24]^. The same number of *IPT* genes are in the *Populous* genome^[29]^, and *PagIPT5a* and *PagIPT5b* are found to be highly expressed in roots and have an opposite expression pattern with *PagARF3.1* during AR formation. The transcript abundance of *PagIPT5a* and *PagIPT5b* were significantly reduced in *PagARF3.1* OE and increased in its RNAi lines. In addition, Y1H analysis showed that PagARF3.1 can directly interact with the promoter regions of *PagIPT5a* and *PagIPT5b*. These results suggest that *PagARF3.1* regulates AR formation through down-regulating *PagIPT5a* and *PagIPT5b*. It has been observed that during the *de novo* shoot regeneration of *Arabidopsis*, *ARF3* reduced cytokinin biosynthesis via physically interacting with the promoter of *AtIPT5* to suppress cytokinin biosynthesis^[22]^. Recently, the auxin-induced expression of *ARF3* has been found to directly and indirectly repress *IPT* and *LONELY GUY* (*LOG*) expression, respectively, to regulate floral meristem determinacy^[23]^. These results demonstrate that the ARF3-IPT5 module represents an evolutionarily conserved mechanism for auxin-cytokinin crosstalk, coordinating diverse developmental processes across species.

## Conclusions

This study showed that *PagARF3.1* is expressed in AR tips, the pericycle cells, early root primordia, and outgrowing roots. Plants with reduced expression of *PagARF3.1* showed delayed AR formation and reduced root biomass. Plants with overexpressed *PagARF3.1* had earlier root formation and increased ARs. RT-qPCR analysis demonstrated that the transcriptional abundance of both *PagIPT5a* and *PagIPT5b* were affected by the manipulation of PagARF3.1. Y1H results showed that PagARF3.1 can bind directly to the promoter regions of *PagIPT5a* and *PagIPT5b*. The present study highlights that PagARF3.1 is a positive regulator of AR formation and demonstrates its critical role in coordinating auxin-cytokinin crosstalk during root development.

## SUPPLEMENTARY DATA

Supplementary data to this article can be found online.

## Data Availability

All data generated or analyzed during this study are included in this published article and its supplementary information files.
