# Supplementary material for: PagARF3.1 promotes adventitious root formation by repressing IPT-mediated cytokinin biosynthesis
Source: For Res (Fayettev). 2025 Aug 29;5:e018. doi: 10.48130/forres-0025-0018 (PMC12442033; doi:10.48130/forres-0025-0018)
Supplement: Supplementary file 1 — Supplementary data to this article can be found online. [file FR-2025-5-0018-Supplementary.zip › 10.48130_forres-0025-0018-Suppl-FigureS2.pdf]

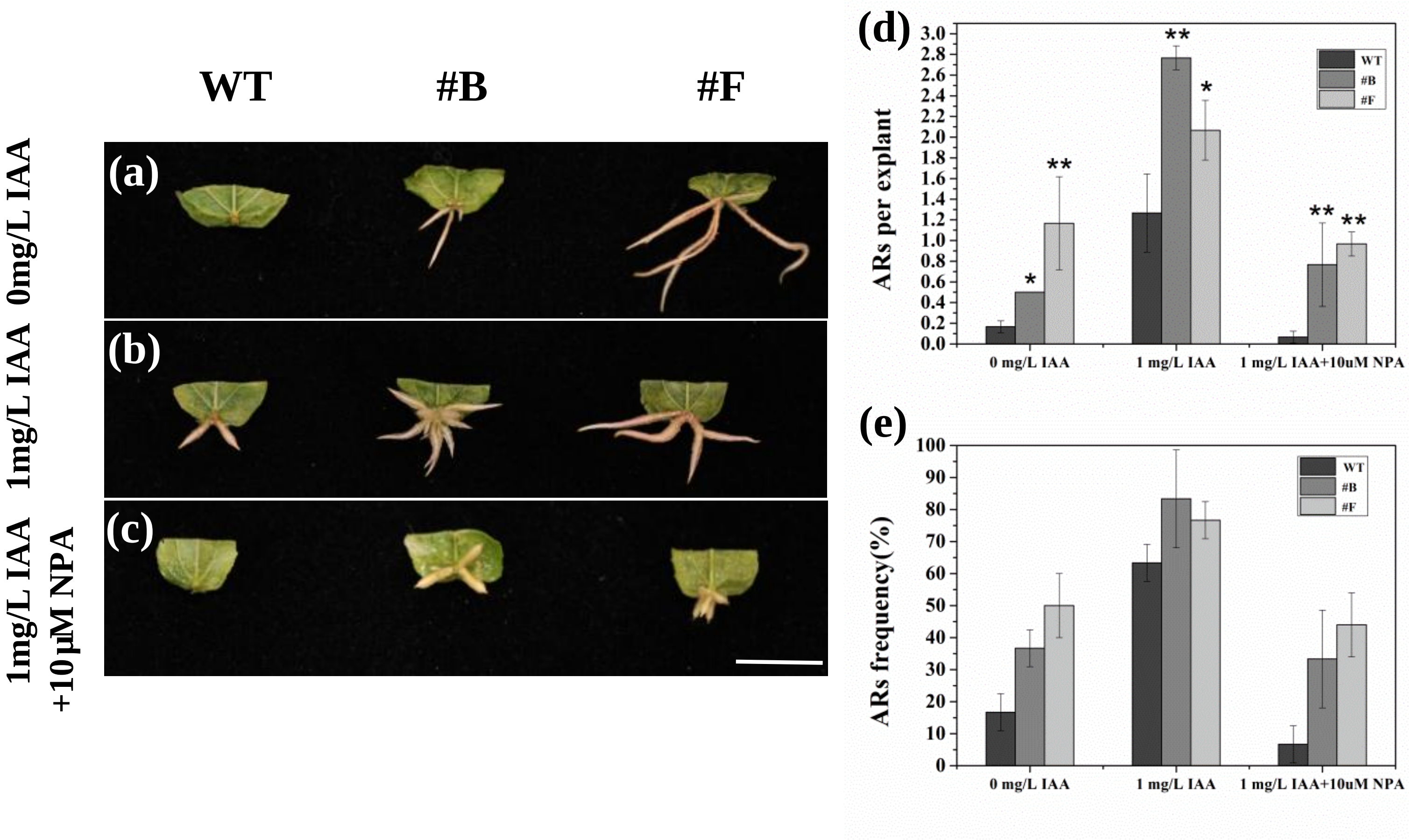

Fig. S2 *PagARF3.1* promotes AR formation via auxin pathway.

Comparison of AR formation from leaf explants between the WT and the overexpression line (#B, #F) of *PagARF3.1* treated with (a) 0 mg/L IAA, (b) 1 mg/L IAA. (c) 1 mg/L IAA+10 μM NPA. Comparison of AR formation (d) frequency, the numbers of AR (e). Significance analysis was performed by Student's t-test (* $p < 0.05$ and ** $p < 0.01$).
