# Supplementary material for: PagARF3.1 promotes adventitious root formation by repressing IPT-mediated cytokinin biosynthesis
Source: For Res (Fayettev). 2025 Aug 29;5:e018. doi: 10.48130/forres-0025-0018 (PMC12442033; doi:10.48130/forres-0025-0018)
Supplement: Supplementary file 1 — Supplementary data to this article can be found online. [file FR-2025-5-0018-Supplementary.zip › 10.48130_forres-0025-0018-Suppl-TableS1.pdf]

Table S1 List of primers used in this study.

Primer	Primer sequence	Application
PagARF3.1F-CDS	GGGGACAACTTTGTACAAAAAAGTTGG AATGATAGATCTTAACACAAC	gene cloning
PagARF3.1R-CDS	GGCGGCCGCACAACTTTGTACAAGAAA GTTGGGTACTAAAAGCACACGATGCCA C	
PagARF3.2F-CDS	GGGGACAACTTTGTACAAAAAAGTTGG A ATGGTGGGTATGATAGATCTCAA	
PagARF3.2R-CDS	GGCGGCCGCACAACTTTGTACAAGAAA GTTGGGTATTAGTATATTCGATAATTTTC A	
proPagARF3.1F	GGGGACAACTTTGTACAAAAAAGTTGG ACAACTTGCGAGCACCTCGAC	Promoter clone
proPagARF3.1R	GGCGGCCGCACAACTTTGTACAAGAAA GTTGGGTAACCCACCATTTTTTGCCTTA	
PagARF3.1-RT-F	CAAGTATCATCACCGTCATCAGT	qRT-PCR
PagARF3.1-RT-R	GCATTGGCTGTCTCCTCTTAG	
PagIPT3-F	GCAAAAGGAGAAGGTTGTGATAGT	
PagIPT3-R	TAGTGACGATGTCAAGTCCTTTGT	
PagIPT5a-F	GGATGTGTCACTACCTCTACTCCA	
PagIPT5a-R	ACATCTCTCACCTCATCAATCAAG	
PagIPT5b-F	GACGTGTCACTCCCAATACTTTAT	
PagIPT5bR	GATCAAACATATCTCTCACCTCATC	
PagIPT6a-F	CACTTCACCCCATTAACATTT	
PagIPT6a-R	CGCTCCCATTATTACGAGAATC	
PagIPT6b-F	AGTTGACTCTCTCCGAGTTTCG	
PagIPT6b-R	CTAGAGCGTGAACAAGGGAGTT	
PagIPT7a-F	GAAGATGTTTGGGACAAGAAGG	
PagIPT7a-R	TTCTTCTGCTTTCTCGTCTTCC	
PagIPT7b-F	GAGATGCTCACTAACTTTCTCCAG	
PagIPT7b-R	CTTTCTCTCTCCATTCAAAGCTAC	
PagIPT9-F	TATTTTGGCCCCTCTGAAACTA	
PagIPT9-R	CCTATCACTGAGTCCATGTCCA	
PagACTIN-F	CACACTGGAGTGATGGTTGG	
PagACTIN-R	ATTGGCCTTGGGGTTAAGAG	
PagARF3.1-AS-F	ACGCGTCGACTAAAGGCATAGGCTTTAATGA ATC	RNAi

PagARF3.1-AS-R	ACGCGGATCCCATTGGCTGTCTCCTCTTAG	
PagARF3.1-S-F	ACGGACTAGTTAAAGGCATAGGCTTTAATGA ATC	
PagARF3.1-S-R	GTCCGAGCTCCATTGGCTGTCTCCTCTTAG	
PagARF3.1-PGADT 7-F	TGGCCATTATGGCCCGGGATGATAGATCTTA ACACAAC	Yeast one hybrid
PagARF3.1-PGADT 7-R	GACATGTTTTTCCCGGGCTAAAAGCACACG ATGCCAC	
3Xcis- F	AATTCTGTCTCTGTCTCTGTCTCGAGCT	
3Xcis- R	CGAGACAGAGACAGAGACAG	
PagIPT5a-pHIS-F	GGAATTCATTGATCGAAGTTCATGTGATG	
PagIPT5a-pHIS-R	CGAGCTCTTATACTGTCATGACTTTGAAGAA T	
PagIPT5b-pHIS-F	GGAATTCGCAATTGTTACTTGTTAGCATGTC	
PagIPT5b-pHIS-F	CGAGCTCAAAGAGGATTCCCCTTGATCTC	
