# Supplementary material for: PagARF3.1 promotes adventitious root formation by repressing IPT-mediated cytokinin biosynthesis
Source: For Res (Fayettev). 2025 Aug 29;5:e018. doi: 10.48130/forres-0025-0018 (PMC12442033; doi:10.48130/forres-0025-0018)
Supplement: Supplementary file 1 — Supplementary data to this article can be found online. [file FR-2025-5-0018-Supplementary.zip › 10.48130_forres-0025-0018-Suppl-FigureS1.pdf]

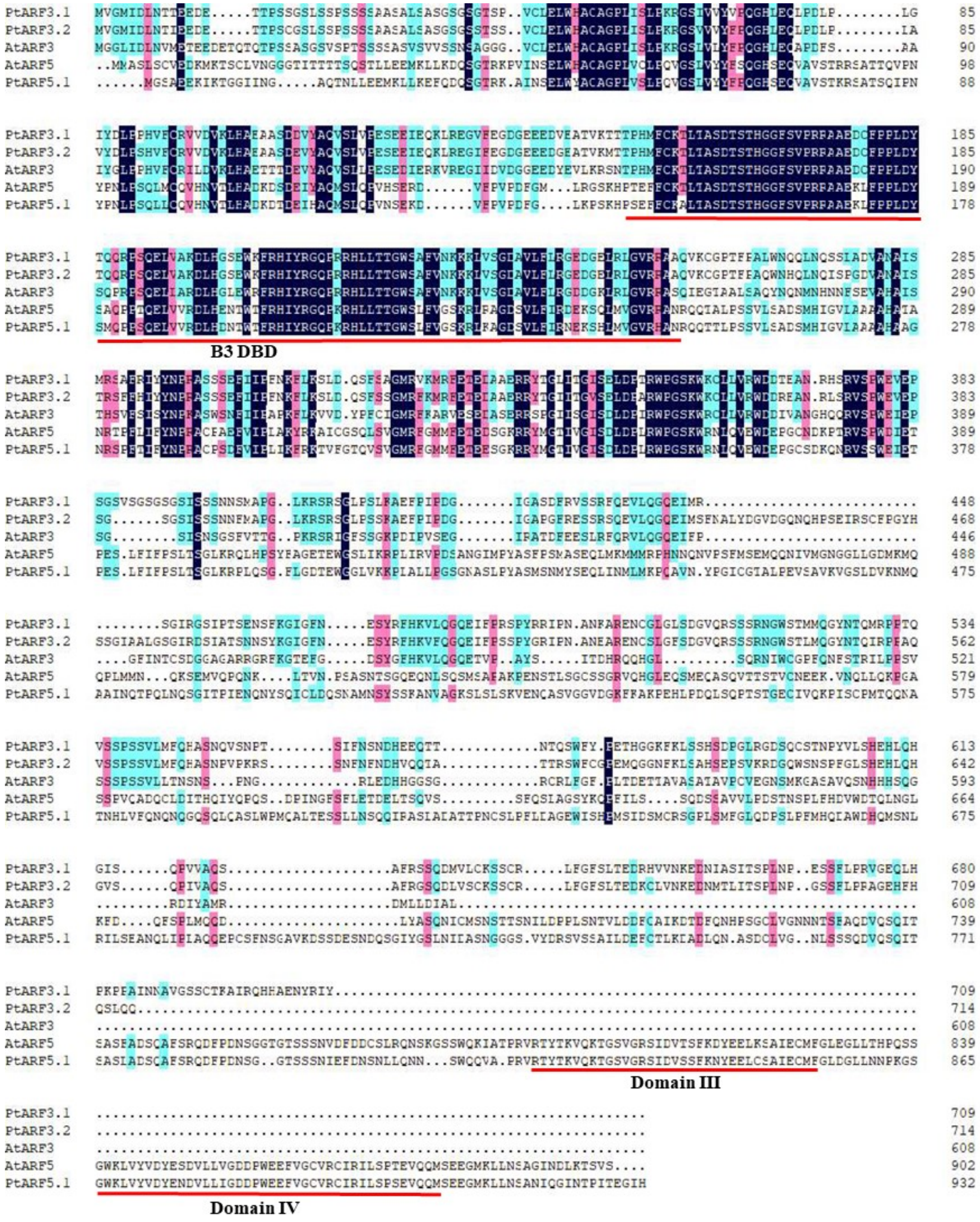

Fig. S1 Sequence alignment of PagARF3.1, PagARF3.2, PagARF5.1, Arabidopsis ARF3 and ARF5. PagARF3.1, PagARF3.2 and ARF3 are lack of the C-terminal dimerization domain (domain III and IV).
