# Supplementary figures and images for: PagARF3.1 promotes adventitious root formation by repressing IPT-mediated cytokinin biosynthesis
Source: For Res (Fayettev). 2025 Aug 29;5:e018. doi: 10.48130/forres-0025-0018 (PMC12442033; doi:10.48130/forres-0025-0018)

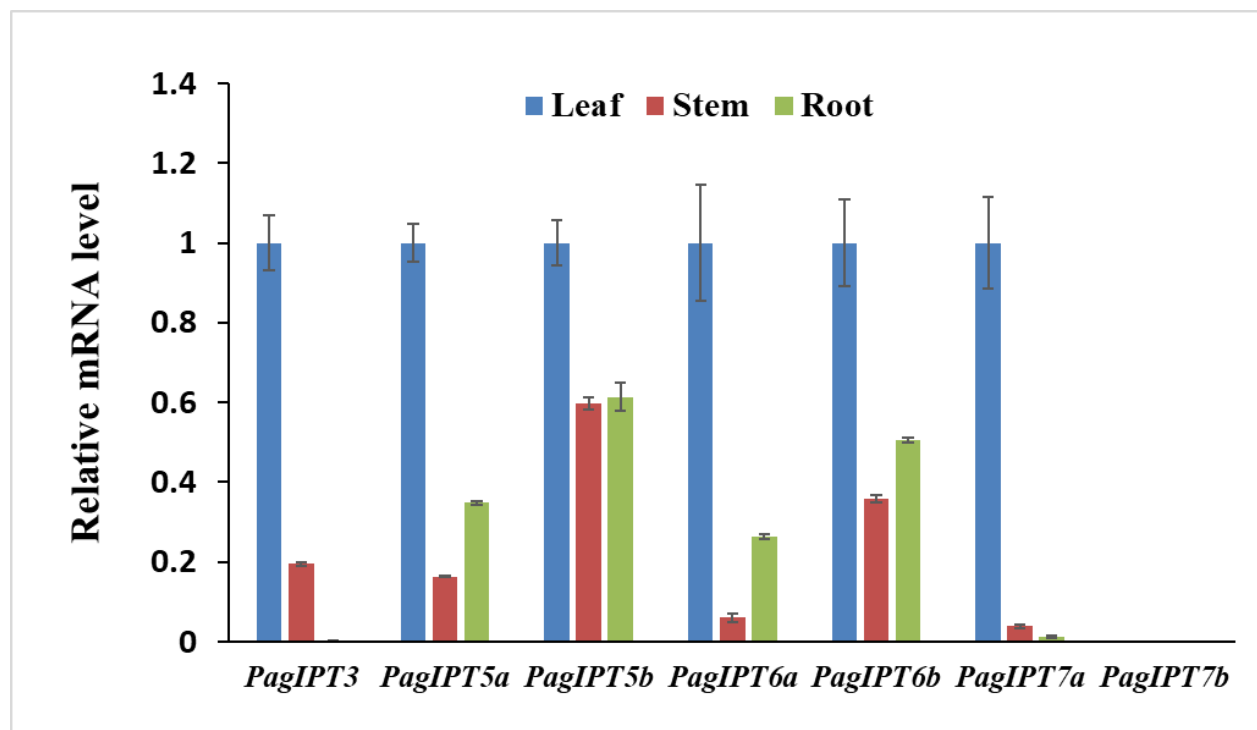

Fig. S3 RT-qPCR analysis of *PagIPT* genes in roots, leaf and stem.

Supplement: Supplementary file 1 — Supplementary data to this article can be found online. [file FR-2025-5-0018-Supplementary.zip › 10.48130_forres-0025-0018-Suppl-FigureS3.pdf]
